# Diversity and Transmission of Gut Bacteria in *Atta* and *Acromyrmex* Leaf-Cutting Ants during Development

**DOI:** 10.3389/fmicb.2017.01942

**Published:** 2017-10-10

**Authors:** Mariya Zhukova, Panagiotis Sapountzis, Morten Schiøtt, Jacobus J. Boomsma

**Affiliations:** Centre for Social Evolution, Department of Biology, University of Copenhagen, Copenhagen, Denmark

**Keywords:** symbiosis, gut microbiota, bacterial transmission, Mollicutes, *Wolbachia*, 16S rRNA sequencing

## Abstract

The social Hymenoptera have distinct larval and adult stages separated by metamorphosis, which implies striking remodeling of external and internal body structures during the pupal stage. This imposes challenges to gut symbionts as existing cultures are lost and may or may not need to be replaced. To elucidate the extent to which metamorphosis interrupts associations between bacteria and hosts, we analyzed changes in gut microbiota during development and traced the transmission routes of dominant symbionts from the egg to adult stage in the leaf-cutting ants *Acromyrmex echinatior* and *Atta cephalotes*, which are both important functional herbivores in the New World tropics. Bacterial density remained similar across the developmental stages of *Acromyrmex*, but *Atta* brood had very low bacterial prevalences suggesting that bacterial gut symbionts are not actively maintained. We found that *Wolbachia* was the absolute dominant bacterial species across developmental stages in *Acromyrmex* and we confirmed that *Atta* lacks *Wolbachia* also in the immature stages, and had mostly Mollicutes bacteria in the adult worker guts. *Wolbachia* in *Acromyrmex* appeared to be transovarially transmitted similar to transmission in solitary insects. In contrast, Mollicutes were socially transmitted from old workers to newly emerged callows. We found that larval and pupal guts of both ant species contained *Pseudomonas* and *Enterobacter* bacteria that are also found in fungus gardens, but hardly or not in adult workers, suggesting they are beneficial only for larval growth and development. Our results reveal that transmission pathways for bacterial symbionts may be very different both between developmental stages and between sister genera and that identifying the mechanisms of bacterial acquisition and loss will be important to clarify their putative mutualistic functions.

## Introduction

Bacterial symbionts of insects can either be vertically transmitted or acquired *de novo* from the environment in every generation. Obligate symbionts are strictly vertically transmitted, but facultative symbionts can be both vertically and horizontally transmitted (Moran et al., 2008; Fisher et al., 2017) and transmission mode appears unrelated to symbiont bacteria having extracellular or intracellular lifestyles (Salem et al., 2015). In solitary insects vertical transmission of symbiotic bacteria can occur via different routes, including the egg’s cytoplasm, the sperm, or substances covering the eggs (Bright and Bulgheresi, 2010; Kaiwa et al., 2014; Watanabe et al., 2014). Complexity culminates in ants and other social insects where vertical transmission can also be achieved through behavioral interaction mechanisms such as larval feeding or grooming (Schmid-Hempel, 1998). This form of vertical transmission at the colony level, based on horizontal transmission between colony members, is unique for social insects (Boomsma et al., 2005) and may facilitate the coexistence and possible co-adaptation of symbiont bacteria and their hosts (Kwong and Moran, 2016). However, different symbionts might be functionally important at different developmental stages of insects, depending on their diet and lifestyle. The question arises, therefore, whether any symbiotic bacteria of ants and other social insects are acquired by direct vertical transmission, and to what extent indirect vertical transmission via nestmates involves the larval stage or not. For symbionts where it does, it might require the evolution of special mechanisms to transmit bacteria during the intricate process of metamorphosis, when extensive cell death occurs in most tissues, including the gut, before a completely restructured adult emerges (Gilbert et al., 2005; Wu et al., 2006; Parthasarathy and Palli, 2008).

Among the social insects, the diversity and transmission of gut bacterial symbionts across developmental stages has been studied in honeybees, termites, and several ant species. It has become clear that larvae of the honeybee *Apis mellifera* have very few or no bacteria in their gut, so that adults need to acquire their gut microbiome through interactions with older nestmates or hive material after emergence (Gilliam, 1971; Gilliam and Prest, 1987; Martinson et al., 2012). The diversity of bacteria in the guts of adult honeybees remains low, as expected for insects with specialized diets (Martinson et al., 2011; Yun et al., 2014), and most of the dominant bacterial species have now been shown to be specialized mutualists with roles in diet supplementation or disease defense (Engel et al., 2012; Kwong and Moran, 2016). In contrast, larvae of the omnivorous fire ant *Solenopsis invicta* digest solid food, turning it into a liquid that workers and queens can ingest, which likely explains that the diversity of larval gut bacteria depends on the geographical location and the food consumed by the colony (Lee et al., 2008; Lee and Hooper-Bui, 2012). A prominent example of direct vertical transmission occurs in *Camponotus* carpenter ants, where transovarially transmitted *Blochmannia* endosymbionts persist in specialized cells that are intercalated in the midgut tissue of all developmental stages (Sauer et al., 2002; Stoll et al., 2010). *Wolbachia* bacteria are endosymbionts present in the guts and other tissues of many insects (Pietri et al., 2016). They are widespread among ant species (Wenseleers et al., 1998) and maternally transmitted by default, as horizontal transfer appears to be exceedingly rare and operates at evolutionary rather than ecological time scales (Shoemaker et al., 2000; Viljakainen et al., 2008; Frost et al., 2010; Martins et al., 2012).

Leaf-cutting ants of the genera *Atta* and *Acromyrmex* have even more specialized diets than honeybees, because they specialize on cultivating a basidiomycete fungal symbiont that provides the only source of nutrition for the larvae. Adult workers also ingest plant sap while cutting leaves and they ingest juice from fallen fruits (Quinlan and Cherrett, 1979), but this does not appear to have increased the diversity of their gut microbiomes. Recent studies have shown that the diversity of the gut bacterial community of adult workers of *Acromyrmex echinatior* is as low as in honeybee workers and mainly composed of a handful of *Wolbachia*, Mollicutes, and Rhizobiales bacteria, whereas gut microbiome diversity in *Atta cephalotes* workers, including Mollicutes, and Rhizobiales, is only slightly higher (Sapountzis et al., 2015; Sapountzis et al., in revision). The *Wolbachia* of *Acromyrmex* are found widespread in the worker bodies and are very unlikely to be reproductive parasites (Andersen et al., 2012). The Rhizobiales form biofilms in the adult worker hindgut and appear to have nitrogen acquisition or preservation functions (Sapountzis et al., 2015), whereas the putative function of the Mollicutes remains to be clarified. There is no indication that Mollicutes bacteria living in *Acromyrmex* and *Atta* leaf-cutting ants are pathogens because they are abundantly present in healthy ant workers.

In other insects, the presence of symbiotic bacteria in specific developmental stages has been used to narrow down the highly diverse potential spectrum of putative functions, which include supplementation of essential nutrients, defense against pathogens, and detoxification of poisonous compounds (Engel and Moran, 2013; Vigneron et al., 2014; Ben-Yosef et al., 2015). In the present study we analyze the abundance and composition of the bacterial community in larval, pupal, and adult guts of two Panamanian leaf-cutting ants, monitor mechanisms of acquisition and changes in bacterial abundance across developmental stages, and discuss possible impacts of the presence or absence of gut bacteria on the health of the ant hosts.

## Materials and Methods

### Ant Collection and Maintenance

Ant colonies were collected in Gamboa, Republic of Panama, in 2014. We used four *Acromyrmex echinatior* colonies (Ae711, Ae712, Ae715, and Ae717) and three *Atta cephalotes* colonies (AcW, AcCr, and AcCa) for sampling workers and brood immediately after collection in the field. Specimens belonged to three developmental stages: actively feeding larvae with clearly visible (under a stereoscope) guts, mature (brown) pupae close to hatching, and adult workers taken either from the top of the fungus garden or foraging around it. For *Acromyrmex*, we collected large workers and for *Atta* media workers of comparable size, of which individual guts were dissected in Gamboa and washed in sterile phosphate-buffered saline (PBS, pH 7.4), transferred to 2 ml screw cap tubes, and stored at -80°C until DNA extraction. For subsequent experiments in the lab to confirm bacterial transmission patterns, we sampled similar specimens from three other *A. echinatior* (Ae322, Ae360, and Ae507) and four other *A. cephalotes* (Ac2012-2, Ac2012-3, Ac2012-4, and Ac19BB) colonies maintained in rearing rooms in Copenhagen (since 1996–2012) at ca. 25°C and 70% relative humidity and fed with bramble leaves, fruit fragments and dry rice. Before dissections, individuals of all developmental stages (including eggs) were always surface-sterilized by submerging them into 70% ethanol and then 2.5% bleach.

To monitor transmission of gut bacteria in real time, we used three of the *A. echinatior* colonies (Ae322, Ae360, and Ae507) and two of the *A. cephalotes* colonies (Ac2012-2 and Ac2012-3) to isolate brood and workers. The infection status of workers was repeatedly confirmed with PCR [presence of known Mollicutes Operational Taxonomic Units (OTUs) *EntAcro1* or *EntAcro2*, see Results; Sapountzis et al., 2015] before the start of the experiment and the following combinations of brood and workers were setup to monitor different transmission patterns: Ae360 pupae + Ae360 workers (transmission toward brood within a single colony where workers were infected with *EntAcro1*), Ae507 pupae + Ae322 workers [transmission across colonies with different Mollicutes infection; *EntAcro1* and weak *EntAcro2* (pupae) versus *EntAcro2* and weak *EntAcro1*(workers)], Ac2012-2 pupae + Ac2012-3 workers and Ac2012-3 pupae + Ac2012-2 workers (transmission across colonies with similar double Mollicutes infection; *EntAcro1* and weak *EntAcro2*). Mature pupae were isolated from colonies and put in large Petri dishes together with fungus garden material and workers from paired colonies. Petri dishes were checked daily for the presence of newly hatched ants and such callows were marked with paint dots on their gasters. After eclosion, callows were together for 1 day with workers from the paired colony to improve the survival of hatched callow workers, which mostly died when they were kept in isolation. Then callows were transferred to a second Petri dish either alone or accompanied by three major and three minor workers from the other colony for 21 days. Ants of both groups were fed with aqueous 10% sucrose solution throughout the experiment and five guts per colony were dissected for pupae and callows both on the first and on the 21st day.

### DNA Extraction and Illumina Miseq Sequencing

DNA was extracted from frozen gut samples of field and lab colonies using the DNeasy blood and tissue kit (Qiagen) according to the manufacturer’s instructions, with an extra step at which glass beads of 0.5 mm were added to lysis buffer and vortexed for 30 s. All samples were eluted using 100 μl of AE elution buffer, after which the extracted DNA from field colonies was sent to the Microbial Systems Laboratory at the University of Michigan for library preparation and 16S Miseq Illumina sequencing as described in Kozich et al. (2013). Sequences of the V4 region of the 16S rRNA genes were amplified using 515F and 806R primers. Amplification was performed using AccuPrime Taq DNA polymerase with high fidelity using the following protocol: 1 cycle at 95°C for 2 min, 30 cycles at 95°C for 20 s, 55°C for 15 s and 72°C for 5 min, and then 1 cycle at 72°C for 10 min. When this standard procedure did not provide enough PCR product for sequencing (larvae, pupae, and some workers of *A. cephalotes*), touchdown PCR was performed under the following conditions: 1 cycle at 95°C for 2 min, 20 cycles at 95°C for 20 s, the annealing temperature ranging from 60°C to 55°C (decreasing 0.3°C every cycle) for 15 s and 72°C for 5 min, then 20 cycles with the same conditions but at a constant 55°C annealing temperature, and finally 1 cycle at 72°C for 10 min. Data were deposited in GenBank (Bioproject PRJNA383138).

### PCR and Droplet Digital PCR (ddPCR)

To check if Mollicutes are present in different developmental stages of ants from lab colonies, we used 16S rRNA gene-specific primers for *EntAcro1* and *EntAcro2* (Supplementary Table S1). To detect *Wolbachia*, we used the *wsp*-specific primers (Braig et al., 1998) and the following PCR conditions: denaturation for 3 min at 94°C, followed by 35 cycles of 30 s at 94°C, 30 s at the relevant annealing temperature (see Supplementary Table S1) and 30 s at 72°C, and a 7-min final extension at 72°C.

For bacterial 16S rRNA gene quantification we used the same DNA samples of guts from field colonies as for Illumina sequencing and a well-established protocol (Sze et al., 2014, see primers in Supplementary Table S1). The bacterial 16S rRNA gene copy load was normalized to the number of ants’ gut cells in the sample using primers for the single-copy gene *tbp* (TATA-box-binding protein, Nygaard et al., 2016, Supplementary Table S1). The amplification process was performed in a Bio-Rad’s S1000 thermal cycler with the following protocol: 1 cycle at 95°C for 5 min, 40 cycles at 95°C for 15 s and 60°C for 1 min, 1 cycle at 4°C for 5 min, and 1 cycle at 90°C for 5 min, all at a ramp rate (heating change rate) of 2°C/s. Two types of negative controls were used for both experiments: water and non-template DNA extracted from a fungus garden of *A. echinatior*, which was reared for three passages on PDYA medium with 15 mg/l of tetracycline and 12 mg/l of streptomycin (Kooij et al., 2015). The negative control 16S bacterial load values never exceeded the range of values described previously for negative controls (Sze et al., 2014) and were below the range of values of our DNA samples. Fluorescence thresholds were chosen manually based on negative control experiments (Supplementary Table S1). The 16S rRNA gene copy number per host cell was calculated as the ratio of 16S rRNA copies per half of the *tbp* copy number, taking into account that there are two gene copies of *tbp* per diploid host cell. Similar procedures were performed to quantify Mollicutes density in transmission experiments with 16S rRNA gene-specific primers for *EntAcro1* and *EntAcro2* (Sapountzis et al., 2015, Supplementary Table S1), except that two additional steps were added for PCR: 1 cycle at 95°C for 5 min, 40 cycles at 95°C for 15 s, 40 s at the annealing temperature (Supplementary Table S1) and 40 s at 72°C, 1 cycle at 4°C for 5 min, and 1 cycle at 90°C for 5 min, all at ramp rate of 2°C/s.

### Data Analyses

To compare bacterial 16S rRNA gene copy densities across different samples, we used non-parametric tests in PAST3 because data were not normally distributed (Hammer et al., 2001). *P*-values below 0.05 were considered statistically significant. 16S rRNA gene sequencing data were analyzed using mothur (version 1.36.1, Schloss et al., 2009) and sequences were processed and filtered as described in the standard operating procedure (SOP) (Kozich et al., 2013). Two sets of reads for each sample were combined into contigs using default parameters in mothur, which allowed most sequencing errors to be removed. All sequences with ambiguous bases and lengths above 275 bp were removed. We subsequently aligned filtered sequences to the SILVA v119 database, removed sequences which did not overlap with the same region of the 16S rRNA gene after alignment, and fixed the maximal allowed homopolymer length at 8. Overhangs at both ends of the aligned sequences were filtered out after which we pre-clustered sequences allowing for up to two nucleotide difference between them. We subsequently removed all chimeric sequences. The remaining set of filtered sequences was assigned to taxonomic groups using the Bayesian classifier with a confidence threshold of 60% using the SILVA v119 database. This allowed us to remove all sequences corresponding to gene fragments of chloroplasts, mitochondria, Archaea and eukaryotes and to cluster the retained bacterial sequences into OTUs based on 97% identity cutoff. Rarefaction curves were generated with randomizations of 1000 and increments of 1000 sequences using mothur and plotted in Microsoft Excel 2010. The final OTU table was rarefied at 4448 reads, which reduced the number of OTUs from 1086 to 718 and was used for all downstream analyses. Kruskal–Wallis and *post hoc* Mann–Whitney tests were used to evaluate significance of individual OTU abundance changes between different developmental stages of the ants. Inverse Simpson indices were estimated in mothur and plotted in JMP 11 to characterize compositional complexity of bacterial communities. To compare the similarity of bacterial community profiles, we performed ordination analysis [non-metric multidimensional scaling (NMDS)] of Bray–Curtis dissimilarity indices, unweighted and weighted UniFrac distances and permutational multivariate analysis of variance (PERMANOVA, permutation number = 9999) in PAST3. Bray–Curtis indices were also calculated in PAST3, while UniFrac distances were computed in mothur.

To compare Mollicutes density in the transmission experiment, we ran a negative binomial regression model for the ddPCR counts in R (R Core Team, 2016), because negative binomial models show a good fit to overdispersed data. Using a Tukey contrast matrix and Bonferroni correction, *post hoc* pairwise tests were run to determine significant differences across four sampling groups (“pupae,” “callows+workers on day 1,” “callows + workers on day 21,” “isolated callows on day 21”) in each experiment with combination of colonies (Ae360 + Ae360, Ae507 + Ae322, Ac2012-2 + Ac2012-3, Ac2012-3 + Ac2012-2; see above for details about these combinations). If there were many zero counts, zero-inflated, hurdle and negative binomial regression models were tested and the Akaike’s Information Criterion (AIC) was calculated for each of them to select the most appropriate model, which always turned out to be a negative binomial or hurdle model. To compensate for the complete separation problem in some colonies where concentrations of bacteria in pupae were zero for all individuals, we ran binomial-response generalized linear models using the bias-reduction method developed by Firth (1993). In this procedure, we first compared the “pupae” to the other three groups (“callows+workers on day 1,” “callows+workers on day 21,” “isolated callows on day 21”) to investigate whether the pupal stage differed from the other three groups by a presence/absence parameter (1/0), and we then removed the pupal stage from our dataset and ran a negative binomial or hurdle model, as described above.

### Microscopy

For fluorescence *in situ* hybridization (FISH), five ant workers, larvae and pupae from *A. echinatior* colony Ae507 and *A. cephalotes* colony 2012-2 were dissected in PBS and their guts placed in 4% paraformaldehyde overnight. For the permeabilization, deproteinization, and hybridization, we followed a previously described protocol (López-Madrigal et al., 2013). For the hybridization step, we used 0.75 μg/μl specific labeled probes targeting bacteria belonging to the class Mollicutes (order Entomoplasmatales) and *Wolbachia-*specific probes (Supplementary Table S1). As negative controls, we used water and reverse probes for Mollicutes (Supplementary Table S1). As positive control, we used the universal bacterial probe EUB388 (Amann et al., 1990) for the pupal stage, because there was no interfering autofluorescence coming from food particles in the gut. The samples were inspected and photographed using a Zeiss LSM 710 confocal microscope equipped with ZEN 2009 software and a Leica TCS SP2 microscope. We considered a signal as being specific when it was absent from the negative controls and colocalized with blue spots produced by simultaneous DAPI staining of bacterial DNA.

For transmission electron microscopy (TEM), larvae, pupae, and large workers of *A. echinatior* colony Ae507 and media workers of *A. cephalotes* colony 2012-2 were dissected in 0.01M phosphate buffer (pH 7.4), and guts were fixed in 2.5% glutaraldehyde (Sigma) in 0.1 M sodium cacodylate buffer (pH 7.4) for 2.5 h. This was followed by washings in the same buffer and postfixation in 1% OsO4 for 1 h, after which samples were placed in a 1% aqueous solution of uranyl acetate and left at 4°C overnight. Samples were then dehydrated in an ethanol series and acetone and embedded in Spurr low-viscosity resin (Ted Pella Inc.). Ultrathin sections of 60–70 nm thickness were stained with uranyl acetate and Reynolds lead citrate and examined with a transmission electron microscope (JEM 1010, JEOL).

## Results

### Quantitative Analysis of Bacterial Density

**FIGURE 1:**
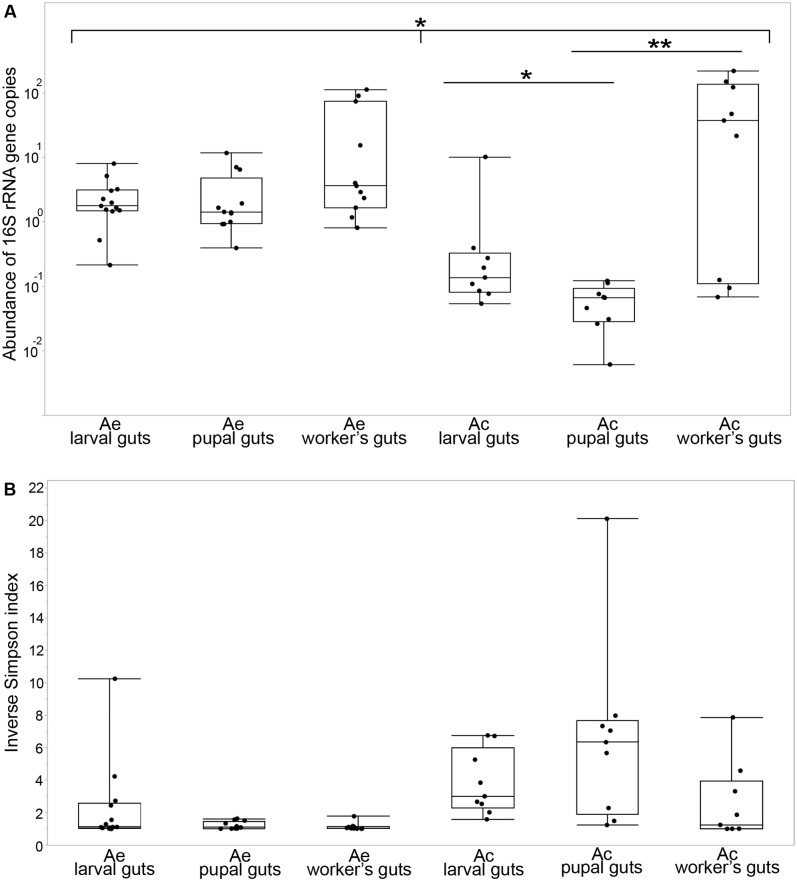
Density and diversity of the gut bacterial communities across the different developmental stages of *Acromyrmex echinatior* (Ae) and *Atta cephalotes* (Ac) leaf-cutting ants. **(A)** Plotted are densities of bacteria (relative abundances of bacterial 16S rRNA gene copies per host cell measured with ddPCR) in the guts of individual larvae, pupae, and adult workers sampled from field colonies. Bars above plots highlight significant differences based on Kolmogorov–Smirnov test for equal distributions between two species and Kruskal–Wallis and *post hoc* Mann–Whitney tests for developmental stages within each species: ^∗^*p* ≤ 0.05, ^∗∗^*p* ≤ 0.01. **(B)** Each point represents the inverse Simpson index of bacterial alpha diversity for a sample collected from an individual (larva, pupa, or adult). Central lines of boxplots are medians, the upper and lower limits of boxes are 75th and 25th quantiles and whiskered bars are minimal and maximal values.

Densities of bacterial 16S rRNA gene copies (**Figure 1A**) were differentially distributed among the two leaf-cutting ant species (Kolmogorov–Smirnov test, *p* = 0.0001). In *Acromyrmex*, there was no significant difference among the three developmental stages (Kruskal–Wallis test, *p* = 0.096), but there was a significant difference among the same developmental stages in *Atta* (Kruskal–Wallis test, *p* = 0.0013; *post hoc* Mann–Whitney test: larvae-pupae: *p* = 0.0102, pupae-workers: *p* = 0.002). Gene copy numbers were similar in larvae and pupae of *A. echinatior*, with median densities of 1.79 and 1.43 copies per host cell, respectively, but less in *Atta* larvae (with a median of 0.14) and the lowest density in *Atta* pupae (0.067 copies per cell). The highest bacterial densities were observed in workers of both species (median 3.65 copies per cell in *A. echinatior* and 37.7 in *A. cephalotes*) and without a significant difference between workers of these two species (Mann–Whitney test, *p* = 0.59). However, bacterial cell numbers were always bimodally distributed across worker samples, with numbers ranging from 0.07 to 222 bacteria per host cell in *A. cephalotes* and from 0.82 to 114 in *A. echinatior*.

### Community Composition of Gut Bacteria Based on 16S rRNA Miseq Sequencing

**FIGURE 2:**
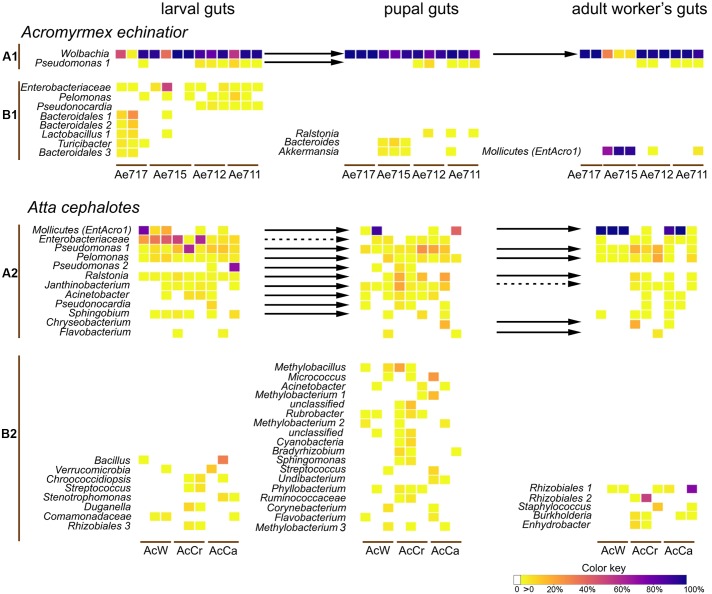
The relative abundances of the main OTUs obtained from the individual gut samples of four *A. echinatior* colonies and three *A. cephalotes* colonies. The top heatmaps for each species **(A1,A2)** include OTUs that were present in at least two developmental stages (connected by arrows when both prevalences exceeded 3%), and the bottom heatmaps **(B1,B2)** give the stage-specific OTUs that were present in at least two individuals (and exceeded 3% relative abundance in at least one individual). Solid arrows designate transitions of bacteria between developmental stages without substantial change in relative abundance while dashed arrows are bacterial transmissions with significant decreases of relative abundance in Mann–Whitney tests (*p* < 0.05).

A total of 31 bacterial classes and 470 OTUs (97% sequence identity cut-off) were identified across the gut communities of *A. echinatior* (12 classes were present in abundances exceeding 1% in at least one individual) and 32 bacterial classes and 374 OTUs were found across the gut communities of *A. cephalotes* (21 exceeding the 1% abundance threshold, Supplementary Table S2). The rarefaction curves approached asymptotes indicating that sufficient sampling depth was achieved for all samples (Supplementary Figure S1). The diversity of bacteria was very low in *Acromyrmex* pupae and adults (inverse Simpson indices close to 1, **Figure 1B**), whereas *Acromyrmex* larvae harbored a higher bacterial diversity (mean inverse Simpson index = 2.3; range 1.01–10.25). Gut bacterial diversity in *Atta* appeared to be very different, with highly diverse pupal gut microbiomes (mean inverse Simpson index = 6.6; range 1.24–20.11) and moderately high diversity in both larvae (mean = 3.82; range 1.5–6.76) and adults (mean = 2.55; range 1.00–7.87) (**Figures 1B**, **2**). Diversity measures were negatively correlated with overall bacterial abundance in *A. cephalotes* (Kendall’s rank correlation, τ = -0.56, *p* = 0.00002), but not in *A. echinatior* (τ = -0.14, *p* = 0.24), suggesting the presence of dominant bacterial OTUs (when inverse Simpson index close to 1) leads to an increase of overall bacterial abundance in *A. cephalotes*, but not in *A. echinatior* (where a dominant OTU was present in almost all individuals analyzed). Overall, nMDS plots generated from a Bray–Curtis dissimilarity matrix revealed two non-overlapping clusters for *A. echinatior* and *A. cephalotes* (Supplementary Figure S2A) and all performed statistical tests of community structure (PERMANOVA) showed a highly significant difference in OTU composition and relative abundances between the two leaf-cutting ant species (Supplementary Table S3).

In *A. echinatior* the most prevalent OTU was a *Wolbachia*, comprising an average of 76.0, 91.6, and 74.9% of the gut bacteria in larvae, pupae and workers, respectively, whereas the most abundant OTU in *A. cephalotes* belonged to the Mollicutes and reached average abundances of 17.2, 21.6, and 81.3% in the guts of larvae, pupae and workers, respectively. However, this only applied to two colonies because the third colony had low (<0.05%) infections in only two individuals and was thus considered to be Mollicutes-uninfected (**Figure 2**). We confirmed that this abundant OTU was identical to the one we identified as *EntAcro1* across workers from six species of Panamanian *Atta* and *Acromyrmex* leaf-cutting ants (Sapountzis et al., 2015; Sapountzis et al., in revision). One of the four colonies of *A. echinatior* (Ae715) also had high gut infection levels with *EntAcro1* (on average 85.5% of all reads, **Figure 2**).

In larvae of *A. echinatior*, several OTUs in addition to the dominant *Wolbachia* OTU reached relative abundances of more than 10% in at least one individual (Enterobacteriaceae, *Lactobacillus* 2, Bacteroidales 1 and 2) and other OTUs hovered between 3 and 10% in several individuals [*Pseudomonas*, *Pelomonas*, *Pseudonocardia* (99% identical to the Ps1 and Ps2 *A. echinatior* symbiont; Andersen et al., 2013), *Lactobacillus* 1, *Turicibacter*, Bacteroidales 2] (**Figure 2**). Larvae of *A. cephalotes* had a relatively high abundance of Enterobacteriaceae (25.6% on average across all sequence reads), *Pseudomonas* 1 (12.7% on average) and *Pseudomonas* 2 (69.8% in one individual). *Atta* guts also harbored rather many OTUs present in several individuals with abundances below 10% (*Pelomonas*, *Ralstonia*, *Janthinobacterium*, *Acinetobacter*, *Sphingobium*) and with abundances of more than 10% but occurring in just one sample (*Lactobacillus* 2, Pseudonocardiaceae, Bacteroidales, *Bacillus*, *Bacteroides*, *Ruminococcus*, Verrucomicrobia, Prevotellaceae). Only OTUs belonging to the Enterobacteriaceae and *Pseudomonas* were present in both *A. echinatior* and *A. cephalotes* larvae.

Pupal gut communities of *A. echinatior* were once more dominated by *Wolbachia*, but also included a *Pseudomonas* OTU that was found in the preceding larval stage. All pupal guts of colony A715, which was highly infected with *EntAcro1* in the adult stage, had OTUs that were not identified in other stages of development (*Bacteroides* and *Akkermansia*). In *A. cephalotes*, most prevalent pupal gut OTUs were also present in larval guts (*EntAcro1*, Enterobacteriaceae, *Pseudomonas* 1 and 2, *Pelomonas*, *Ralstonia*, *Janthinobacterium*, *Acinetobacter*, *Pseudonocardia*, and *Sphingobium*), although the relative abundance of Enterobacteriaceae significantly decreased in the pupal stage in comparison to larval guts (Mann–Whitney test, *p* = 0.002). There were also some unique pupal OTUs, which were present in abundances of more than 10% of the total reads in at least one individual (e.g., *Methylobacillus*, *Micrococcus*, and *Methylobacterium*) and that did not appear in other developmental stages (**Figure 2**).

The adult worker gut community of *A. echinatior* was completely dominated by *Wolbachia* and *EntAcro1*, which jointly accounted for 97–99.8% of the total number of reads per individual. Bacterial diversity of gut communities of *A. cephalotes* workers varied depending on the colony’s infection with *EntAcro1*. When colonies were infected by these Mollicutes bacteria, they represented 89–99.9% of all sequence reads for separate individuals. However, there were some *EntAcro1*-uninfected workers, and these uninfected workers had more diverse gut microbial communities, including both specific for this stage OTUs (e.g., two Rhizobiales OTUs, *Staphylococcus*) and OTUs that were present also in larvae and pupae (e.g., *Pseudomonas*, *Pelomonas*). Rhizobiales, *Pseudomonas*, *Pelomonas*, *Staphylococcus*, *Chryseobacterium*, Cyanobacteria, and *Lactococcus* occurred in abundances of more than 10% of all reads at least in one individual. Overall, *Atta* workers had either very few bacteria in their guts and then their microbiomes were relatively diverse, or they had many and then gut bacteria were almost exclusively Mollicutes. When we compared abundances of 16S rRNA gene copy numbers for workers with proportional presences of Mollicutes <0.1% and >0.1%, the latter mean absolute number of bacteria was >20 times higher in *Atta*, but only 3.4 times higher, and not significantly so, in *Acromyrmex* (Supplementary Figure S3, Mann–Whitney test, *A. echinatior*: *p* = 0.219, *A. cephalotes*: *p* = 0.019).

### Analyses of Beta Diversity across Developmental Stages

We used a PERMANOVA test to evaluate the similarity of gut communities across the larval, pupal, and adult stages (Supplementary Table S3). For *A. echinatior* we found a significant difference in beta diversity between both developmental stages and the colonies sampled, and also a significant stage × colony interaction in all tests performed (using Bray–Curtis dissimilarities, unweighted and weighted UniFrac metrics). The nMDS plots revealed close similarity between the two colonies with dominant *Wolbachia* infections across all developmental stages (Ae711 and Ae712), whereas all stages of the colony with dominant *EntAcro1* (Ae715) had a different bacterial community, and also the larvae of colony Ae717 with high bacterial diversity and low *Wolbachia* titer stood out (Supplementary Figure S4). For *A. cephalotes* we found a significant difference, both between colonies and developmental stages (Supplementary Figures S5, S6) in all tests performed, but no significant stage × colony interaction was found in tests performed on either Bray-Curtis dissimilarities or weighted UniFrac distances (Supplementary Table S3). Pairwise differences in beta diversity were significant between larvae and workers of *A. cephalotes* (*p* = 0.0291) and between Mollicutes-infected (AcW) and uninfected (AcCr) colonies (*p* = 0.0021) in Bray–Curtis index comparisons, but there was no significant difference between groups in tests with weighted UniFrac distances (Supplementary Table S4).

### Transovarial Transmission for *Wolbachia* But Not for Mollicutes

Assuming that transmission mechanisms of dominant bacteria are similar in field and lab colonies, we tested the presence of *Wolbachia* and Mollicutes in *A. echinatior* and *A. cephalotes* lab colonies by PCR and included also eggs in the analysis (**Table 1** and Supplementary Table S5). *Wolbachia* was always present in worker guts of *A. echinatior*, but never in *Atta. EntAcro1* and *EntAcro2* OTUs were universally found in worker guts of both *A. echinatior* and *A. cephalotes*, either in single or double-infections. *Wolbachia* bacteria were present in the eggs of all *Acromyrmex* colonies and remained present in all developmental stages (Supplementary Table S5). However, the Mollicutes were absent in the eggs of all three *Acromyrmex* and all four *Atta* colonies (**Table 1**). Mollicutes were present in very low abundance in guts of some larvae of both ant species, but were detected just once in the gut of a pupa of *A. echinatior*, which is similar to the pattern observed in the field colonies (Supplementary Table S6). It is noteworthy that Mollicutes were sometimes found in the remaining bodies of dissected pupae from field and lab colonies, even though the guts of the same pupae were most often uninfected (**Table 2** and Supplementary Table S7). Finally, Mollicutes were present in the guts of some, but not all adult workers of both *A. echinatior* and *A. cephalotes*.

**Table 1:** Presence of the two Mollicutes OTUs, *EntAcro1* and *EntAcro2*, in different developmental stages in lab colonies of *A. cephalotes* and *A. echinatior* based on PCR analysis.

Ant colony		Ae322		Ae360		Ae507		Ac2012-2		Ac2012-3		Ac2012-4		Ac19BB	
Eggs		**-**	**-**	**-**	**-**	**-**	n/a	**-**	**-**	n/a	**-**	**-**	**-**	n/a	**-**
Larval guts	1	(+)	**-**	+	**-**	**-**	n/a	**-**	**-**	n/a	**-**	**-**	**-**	n/a	**-**
	2	(+)	**-**	**-**	**-**	+	n/a	**-**	**-**	n/a	**-**	**-**	**-**	n/a	**-**
	3	(+)	**-**	(+)	**-**	(+)	n/a	**-**	**-**	n/a	**-**	**-**	**-**	n/a	**-**
	4	**-**	**-**	**-**	**-**	**-**	n/a	(+)	**-**	n/a	**-**	**-**	**-**	n/a	**-**
	5	**-**	**-**	**-**	**-**	(+)	n/a	**-**	**-**	n/a	**-**	**-**	**-**	n/a	**-**
Pupal guts	1	**-**	**-**	**-**	**-**	**-**	n/a	**-**	**-**	n/a	**-**	**-**	**-**	n/a	**-**
	2	**-**	**-**	**-**	**-**	**-**	n/a	**-**	**-**	n/a	**-**	**-**	**-**	n/a	**-**
	3	**-**	**-**	**-**	**-**	**-**	n/a	**-**	**-**	n/a	**-**	**-**	**-**	n/a	**-**
	4	**-**	**-**	**-**	**-**	**-**	n/a	**-**	**-**	n/a	**-**	**-**	**-**	n/a	**-**
	5	(+)	**-**	**-**	**-**	**-**	n/a	**-**	**-**	n/a	**-**	**-**	**-**	n/a	**-**
Worker guts	1	**-**	+	+	**-**	+	**-**	+	**-**	**-**	**-**	**-**	+	**-**	+
	2	(+)	+	+	**-**	+	**-**	+	**-**	**-**	+	+	+	**-**	+
	3	(+)	+	+	**-**	+	**-**	+	**-**	**-**	+	+	**-**	**-**	+
	4	**-**	+	+	**+**	+	**-**	+	**-**	**-**	(+)	+	+	**-**	+
	5	(+)	+	+	**-**	+	**-**	+	+	**-**	+	+	+	**-**	+
**Bacterial OTU**		*EntAcro1*	*EntAcro2*	*EntAcro1*	*EntAcro2*	*EntAcro1*	*EntAcro2*	*EntAcro1*	*EntAcro2*	*EntAcro1*	*EntAcro2*	*EntAcro1*	*EntAcro2*	*EntAcro1*	*EntAcro2*


*Numbers (1–5) mark individual ant samples. + indicates that bacteria were present, (+) that bacteria were present only in a very low abundance, corresponding to extremely faint bands after agarose gel electrophoresis; - that bacteria were absent, and n/a that samples were not analyzed.*

**Table 2:** Presence of the two Mollicutes OTUs, *EntAcro1* and *EntAcro2*, in *A. cephalotes* pupal guts and the rests of the body of the same individuals.

Pupae of *Atta cephalotes*	Field colonies																				Bacterial OTU
		
		AcCa						AcCr						AcW						
Gut			+	-	-				-	-	-				-	+	-				*EntAcro1*
Rest of the body			+	+	+				-	-	+				+	+	+				
Gut			-	-	-				-	-	-				-	-	-				*EntAcro2*
Rest of the body			-	-	-				-	-	-				-	-	-				

**Pupae of *Atta cephalotes***	**Lab colonies**																				**Bacterial OTU**
		
	**Ac2012-2**					**Ac2012-3**					**Ac2012-4**					**Ac19BB**					

Gut	-	-	-	-	-	-	-	-	-	-	-	-	-	-	-	-	-	-	-	-	*EntAcro1*
Rest of the body	-	+	-	-	+	-	-	-	-	-	+	+	+	+	+	-	-	-	-	-	
Gut	-	-	-	-	-	-	-	-	-	-	-	-	-	-	-	-	-	-	-	-	*EntAcro2*
Rest of the body	-	+	-	-	+	-	-	-	-	+	-	-	-	+	-	-	-	-	-	-	


*Cells refer to individual samples (three replicates for field colonies and five replicates for lab colonies). Samples with infected tissues are marked with +, whereas - indicates no infection.*

**FIGURE 3:**
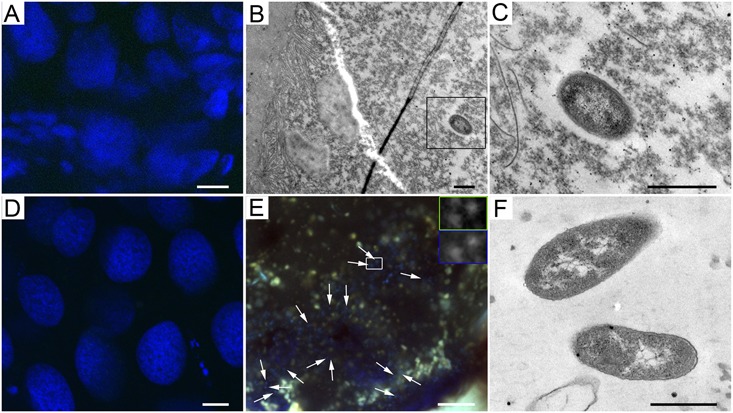
Mollicutes bacteria in *A. cephalotes* across developmental stages. **(A,D)** Absence of Mollicutes in larval **(A)** and pupal **(D)** tissues of *A. cephalotes*. **(B)** Gram-negative bacterium in the lumen of a larval gut of *A. cephalotes.* **(C)** Higher magnification of the bacterium framed in **B**. **(E)** Highly abundant Mollicutes in a worker’s rectum of *A. cephalotes* (arrows point at some of the bacteria). Frames show bacteria stained with a Mollicutes-specific probe (framed green) and DAPI (framed blue) at higher magnification. **(F)** Mollicutes-like bacteria lacking a cell wall in the rectum of *A. cephalotes* worker. Scale bars are 10 μm **(A,D,E)** and 0.5 μm **(B,C,F)**.

Microscopy of the lab colony of *A. echinatior* showed that *Wolbachia* bacteria were present in the epithelial cells of the larval gut (Supplementary Figures S7A,B) and in all pupal gut compartments (crop, midgut, ileum, and rectum), but seldom in the pupal Malpighian tubules (Supplementary Figures S7C–G). Some *Wolbachia* bacteria were in close contact with mitochondria in pupal and adult tissues (Supplementary Figures S7H,I). We could not localize Mollicutes in larval and pupal guts from the lab colony of *A. cephalotes*, confirming that bacterial presence (including Mollicutes) in these stages is very low (**Figures 3A,D**). Nevertheless, there were a few Gram-negative bacteria in the gut lumen of larvae of *A. cephalotes*, mixed with homogenous food (**Figures 3B,C**), suggesting that larvae acquired them with the ingested fungus. However, Mollicutes were highly abundant in the rectum of adult *A. cephalotes* workers supporting our quantitative ddPCR data (**Figure 3E**). The Mollicutes-like bacteria that we observed with TEM lacked a cell wall, which is a unique feature of Mollicutes, and had rod shapes with an average diameter 0.4 μm and a length range of 0.7–1.9 μm (**Figure 3F**).

### Test of the Hypothesis of Social Transmission for Mollicutes

**FIGURE 4:**
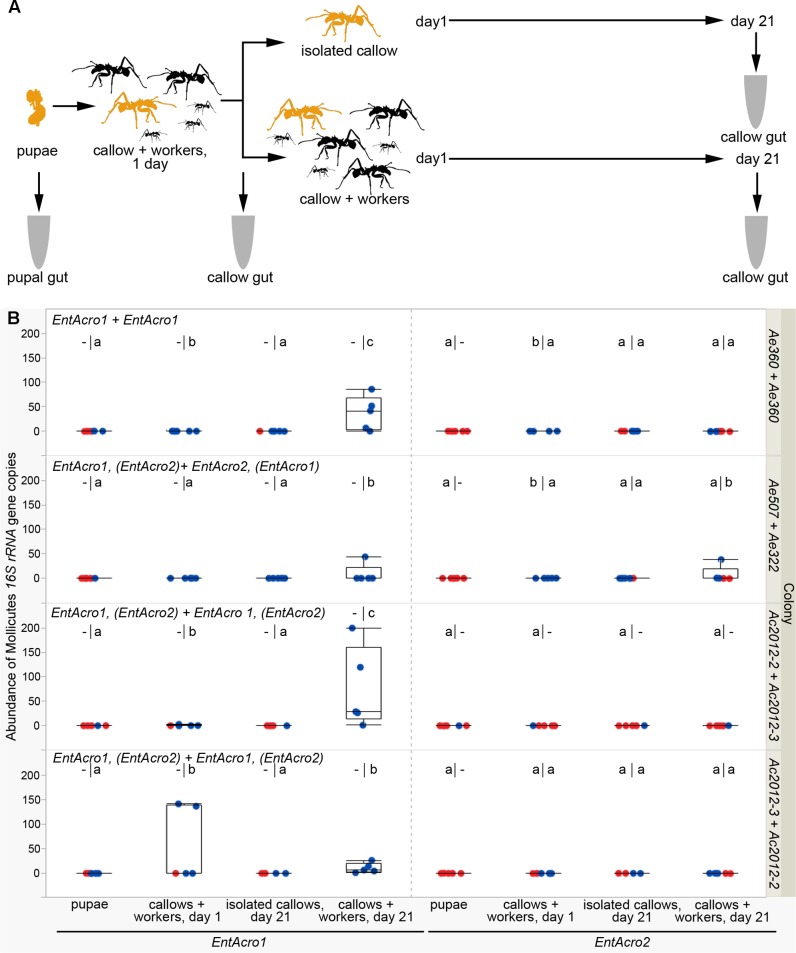
Acquisition of Mollicutes bacteria by workers after hatching. **(A)** Schematic diagram of the experiment. Pupae and newly emerged workers are colored in yellow, while major and minor ants from the paired colony are shown in black. Guts of pupae and callows were sampled for analyses. **(B)** Density of Mollicutes expressed as *16S rRNA* gene copies normalized per gut cell number. The first Mollicutes OTUs above each plots correspond to the known infections of the colony from which pupae were taken and the second (after the +) to the known infections of the nursing workers that interacted with callows emerging from these pupae (in brackets when Mollicutes were not present in all tested workers or were present in low abundances; for more information see Supplementary Table S8). Red dots correspond to bacterial scores of zero, while blue dots are scores above zero. The lower-case letters above each boxplot indicate significant differences between groups resulting from two different statistical tests. On the left side, different letters indicate significant differences (*p* < 0.05) in binomial (1/0 = presence/absence) tests (performed only in colonies where the “pupae” group had all zero scores and this group was omitted from further analysis). On the right side, different letters indicate significant differences (*p* < 0.05) in bacterial densities between groups in *post hoc* pairwise multiple comparisons. – indicates that a corresponding test was irrelevant or could not be performed.

Before the bacterial transmission experiment (**Figure 4A**), we tested Mollicutes infection status in workers from the lab colonies. We found that *EntAcro1* had displaced *EntAcro2* in most colonies that we analyzed (Supplementary Table S8) during a 9 months period that had passed since our previous check (**Table 1**). Measurement of Mollicutes density with sensitive ddPCR technology confirmed that both *EntAcro1* and *EntAcro2* were absent or had very low density in mature pupae (medians below 0.01 16S rRNA gene copies per gut host cell in all colonies, **Figure 4B**). After 21 days in isolation, no workers (*n* = 20, checked for both *EntAcro1* and *EntAcro2*) showed an increase in density of Mollicutes in their guts and often the density was similar to that of pupae, implying that bacteria do not reinfect and multiply in the gut of workers from adjacent tissues after eclosion. In contrast, many of the workers that had been in contact with other workers for 21 days after eclosion, showed a significant increase in Mollicutes density, suggesting that interaction with workers in the absence of fungus garden is enough for Mollicutes transmission between ants from the same or different colonies.

## Discussion

### Diversity and Density of Gut Bacteria during Different Developmental Stages

Like other Hymenoptera, ants undergo complete metamorphosis, in which larvae stop feeding and purge their gut before pupation. During metamorphosis larval gut epithelium undergoes programmed cell death and is replaced by pupal/adult epithelium. In the gut, the pupa stores metabolic wastes as a meconium which is excreted when the adult hatches. The complete remodeling of the gut thus provides great opportunities to change gut bacterial communities across developmental stages, and this flexibility may be especially important for species in which larvae and adults have different diets and lifestyles. So far, very few other studies on social insects have provided evidence for distinct microbial community differences between larval and worker guts. *Enterobacter* and *Pseudomonas*, dominant bacteria of the fungus gardens in both *A. cephalotes* and *A. echinatior* (Aylward et al., 2014), were found in larval guts, but disappeared in workers of *A. echinatior* or remained present only at highly reduced abundances in *A. cephalotes*. Representatives of several bacterial families [Bacillaceae, Burkholderiaceae (*Ralstonia*), Comamonadaceae, Flavobacteriaceae, Moraxellaceae, Sphingomonadaceae] were also observed in the fungus garden of *Atta* species (Suen et al., 2010; Aylward et al., 2012) and bacterial OTUs that we found in larval guts (*Bacillus*, *Ralstonia*, *Pelomonas*, *Chryseobacterium*, *Flavobacterium*, *Acinetobacter*, and *Sphingobium*) belong to the same families. This suggests that the fungus garden plays an important role in supporting the persistence of these bacteria within colonies, but further study will be required to fully understand their beneficial role for larvae or fungus gardens. The few *Pseudonocardia* bacteria that were present in larval guts of *A. echinatior* (with relative abundance ranging from 0.07 to 3.2%) could come from the cuticle of the workers feeding them or from the fungus garden assuming workers would inoculate these as well. Studies of bee species have shown that honey bee larvae harbor both bacteria belonging to the core gut microbiome of adults and other OTUs that potentially originate from nectar sources (Mohr and Tebbe, 2006; Vojvodic et al., 2013), while bumblebee larvae have gut bacteria of adults but in different proportion (Mohr and Tebbe, 2006).

Despite the fact that larvae purge the gut before pupation, bacteria have been found in pupae of insects from various orders: Coleoptera (Delalibera et al., 2007), Diptera (Wong et al., 2011), Lepidoptera (Hammer et al., 2014; Johnston and Rolff, 2015), and Hymenoptera (Brucker and Bordenstein, 2012). In our study, the bacterial load was very low in mature pupae of *A. cephalotes*, implying that metamorphosis resulted in near-complete elimination of gut bacteria, but in *A. echinatior* the bacterial load did not decrease relative to the larval stage and *Wolbachia* accounted for >90% of the overall abundance of pupal gut bacteria. In some insects, bacteria in the pupal gut determine the adult gut microbiota (Johnston and Rolff, 2015), while others acquire gut bacteria after metamorphosis from their food and environment (Engel and Moran, 2013). Our data suggest that adult workers of both leaf-cutting ant species lose most of the bacteria present in the early developmental stages and re-establish the adult gut microbiota after eclosion.

### Mollicutes Social Transmission Patterns

Our results matched the expectation that *Wolbachia* are maternally inherited bacteria, as shown for solitary insects (e.g., Serbus et al., 2008), but Mollicutes were absent in eggs of all leaf-cutting ants that we investigated. Previous studies in the army ant *Eciton burchellii* likewise did not find Entomoplasmatales bacteria in eggs, whereas in the fungus-growing ant *Sericomyrmex amabilis*, Entomoplasmatales were found sporadically (in 4 of 10 eggs from one colony) (Funaro et al., 2011; Liberti et al., 2015). Some larvae of *A. cephalotes* from the field and *A. echinatior* from the lab had Mollicutes in their guts. In army ants, Entomoplasmatales occurred sporadically in larvae of *E. burchellii* and pupae of *Dorylus molestus*, but were absent in most larvae and pupae of other species analyzed (Funaro et al., 2011). In both *Atta* and *Acromyrmex* species workers play a major role in feeding the larvae. In *Acromyrmex subterraneus brunneu*s, workers macerate fungal staphylae by mandibular movements before feeding them to larvae (Camargo et al., 2006), while workers of *Atta sexdens* deposit the fungal mass, prepared with other mouthparts than the mandibles, directly on a larva’s mandibles (Schneider et al., 2000). Although other details may differ, this broad similarity in feeding behavior suggests that transmission of Mollicutes to larval guts can happen through active feeding in both species. Pupal guts of *Acromyrmex* and *Atta* occasionally had Mollicutes, while the remaining part of the body carried these bacteria more often. Previous studies have shown that Mollicutes can be both intracellular and extracellular symbionts in ants, and some bacteria of the same order were also isolated from the hemolymph of a firefly beetle (Tully et al., 1989; Sapountzis et al., 2015). We thus hypothesize that Mollicutes bacteria infect hemolymph and surrounding tissues of leaf-cutting ants during remodeling of the internal anatomy in the pupal stage, but further work will be needed to substantiate this.

In some solitary insects, Mollicutes increase longevity and fecundity of their vector hosts (Ammar and Hogenhout, 2006), but it remains as yet unclear whether and how Mollicutes (*EntAcro1* and *EntAcro2*) are beneficial for *Acromyrmex* and *Atta* leaf-cutting ants. In the adult workers, the Mollicutes were most abundant in the rectum, suggesting that this is the most favorable niche for them. Both *Acromyrmex* and *Atta* also maintain Rhizobiales bacteria, which have nitrogen fixing/preservation functions and form a biofilm adhering to the cuticle in the ileum and rectum of workers (Sapountzis et al., 2015; Sapountzis et al., in revision), but there is no direct evidence that Rhizobiales compete with Mollicutes for host resources in the rectum. Mollicutes are also increasingly documented to be widespread among ant species, with recent studies having demonstrated their presence in workers of *Solenopsis* species, Ponerinae ants, turtle ants, and *Atta texana* (Ishak et al., 2011; Kautz et al., 2013; Meirelles et al., 2016; de Oliveira et al., 2016). Our experiments clearly indicate that Mollicutes are transmitted through social interactions between workers. Social transmission of bacteria has also been shown for other social insects, such as honeybees and bumblebees (Martinson et al., 2012; Billiet et al., 2017). These findings suggest that Mollicutes may be a generally underappreciated lineage of symbionts across the ants that would be interesting for further studies.

### Putative Functions of Bacterial Gut Symbionts in Leaf-Cutting Ants

The unique lifestyle of social insects allows certain types of specialized bacteria to become established and be transmitted within colonies that would not be able to maintain themselves as symbionts of solitary insects. Our results reveal at least two different pathways by which bacterial OTUs can persist within leaf-cutting ant colonies and indicate that unraveling the stages of bacterial acquisition gives useful information about the putative mutualistic functions of symbionts across developmental stages. This is particularly so for the socially transmitted Mollicutes symbionts (*EntAcro1* and *EntAcro2*), which showed enormous variation in abundance both across and within colonies of *A. echinatior* and *A. cephalotes*. Although these bacteria are mostly known as mild pathogens (Meirelles et al., 2016), such antagonistic functions seem highly unlikely for the *EntAcro1* and *EntAcro2* symbionts of leaf-cutting ants because we never observed any disease symptoms in ants that were later shown to carry Mollicutes in high abundances. However, these bacteria are clearly facultative symbionts across the leaf-cutting ants, so further work to clarify their functions will be rewarding.

When we combine the findings of our present studies with earlier results (Frost et al., 2010; Andersen et al., 2012; Sapountzis et al., 2015), it appears that *Wolbachia* is an obligate symbiont throughout the developmental stages of *Acromyrmex* leaf-cutting ants. The symbiotic function of these bacteria remains enigmatic, but their abundant presence and close interaction with mitochondria in the ant cytoplasm corroborate that *Wolbachia* is a highly adapted symbiont and worth further study to clarify its functions both in the larval and adult stages.

In this first systematic study of bacterial symbionts of ant brood, we found that both *Enterobacter* and *Pseudomonas* bacteria were relatively abundant in larval guts and hardly present in adult worker guts. We suggest that these bacteria may be involved in immune priming, and suggest that further work in this direction may be worthwhile. It has been shown that larvae of both social and solitary insects, including carpenter ants *Camponotus pennsylvanicus*, tiger moths *Parasemia plantaginis*, and cabbage semilooper *Trichoplusia ni*, all exhibit immune priming (Freitak et al., 2007; Rosengaus et al., 2013; Mikonranta et al., 2014), making larvae less susceptible to pathogens after both oral exposure to non-pathogenic and pathogenic bacteria and injection with killed pathogenic bacteria.

## Ethics Statement

The research project was conducted on invertebrate species that are not subjected to any specific ethical issue and legislation. The Autoridad Nacional del Ambiente de Panama (ANAM) issued permits for collection and export of live and frozen ant material from Panama to Denmark (2014).

## Author Contributions

MZ and JB carried out the field sample collection, MZ and PS performed the experiments and data analysis. MZ, PS, MS, and JB designed the study, drafted and wrote the manuscript. All authors read and approved the final manuscript.

## Conflict of Interest Statement

The authors declare that the research was conducted in the absence of any commercial or financial relationships that could be construed as a potential conflict of interest.
