# Supplementary material for: Diversity and Transmission of Gut Bacteria in Atta and Acromyrmex Leaf-Cutting Ants during Development
Source: Front Microbiol. 2017 Oct 10;8:1942. doi: 10.3389/fmicb.2017.01942 (PMC5641371; doi:10.3389/fmicb.2017.01942)
Supplement: Supplementary file 2 [file Data_Sheet_1.PDF]

Supplementary material

Diversity and Transmission of Gut Bacteria in *Atta* and *Acromyrmex* Leaf-Cutting Ants during Development

Mariya Zhukova*, Panagiotis Sapountzis, Morten Schiøtt, Jacobus J. Boomsma*

***Correspondence:**

Mariya Zhukova: mariya.zhukova@bio.ku.dk; Jacobus J. Boomsma: JJBoomsma@bio.ku.dk

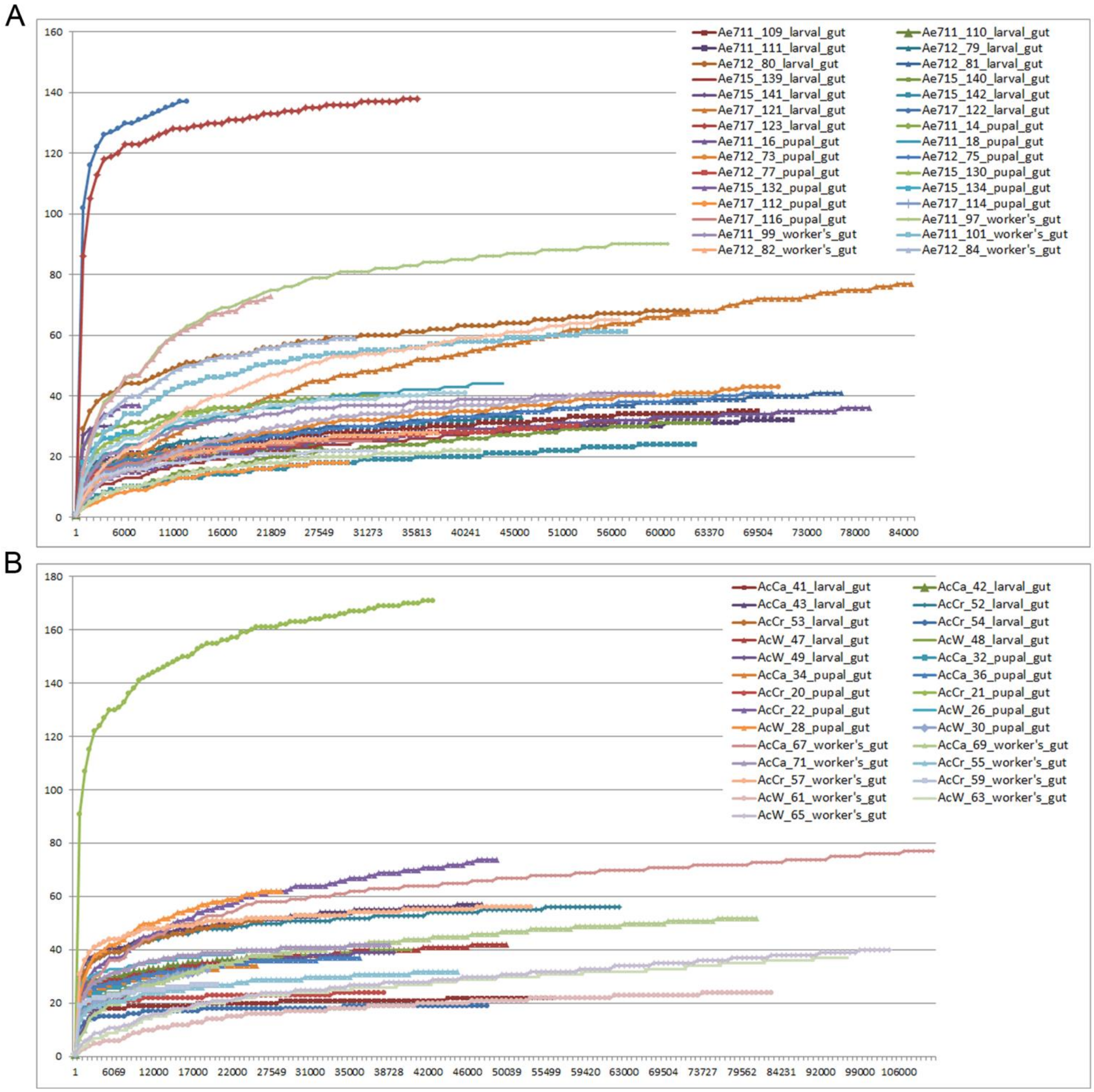

Figure S1. Rarefaction curves for all individual samples of *A. echinatior* (A) and *A. cephalotes* (B). Curves show the number of bacterial OTUs as a function of sequencing depth. Rarefaction analysis was carried out using pseudoreplicate OTU datasets containing between 1 and 112610 sequences and with 1000 repetitions per pseudoreplicate.

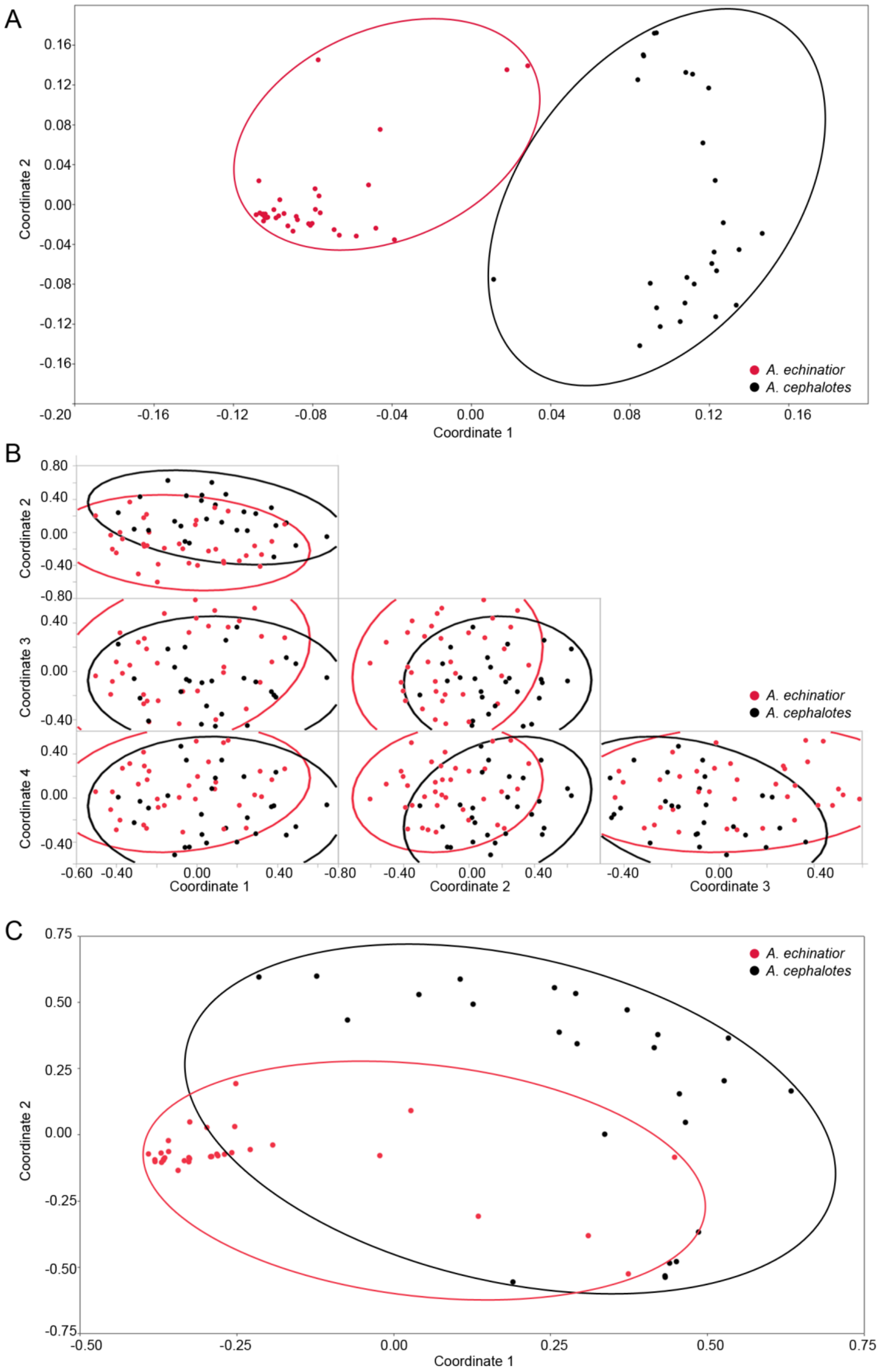

Figure S2. Non-metric multidimensional scaling (NMDS) diagram of gut bacterial communities of *A. echinatior* and *A. cephalotes* based on Bray-Curtis dissimilarity matrix (A), unweighted and weighted UniFrac distances (B and C, respectively).

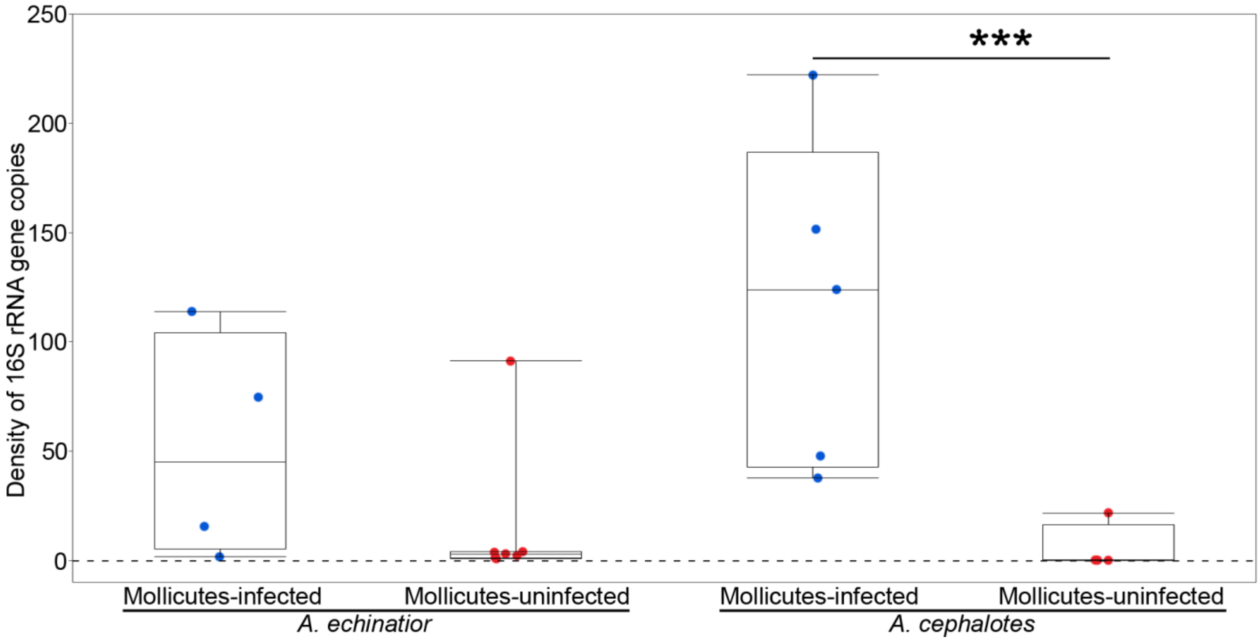

Figure S3. Comparison of the load of bacterial 16S rRNA gene copy number in Mollicutes-infected and Mollicutes-uninfected workers of *A. echinatior* and *A. cephalotes*. Mann-Whitney test: *** = $p \leq 0.05$.

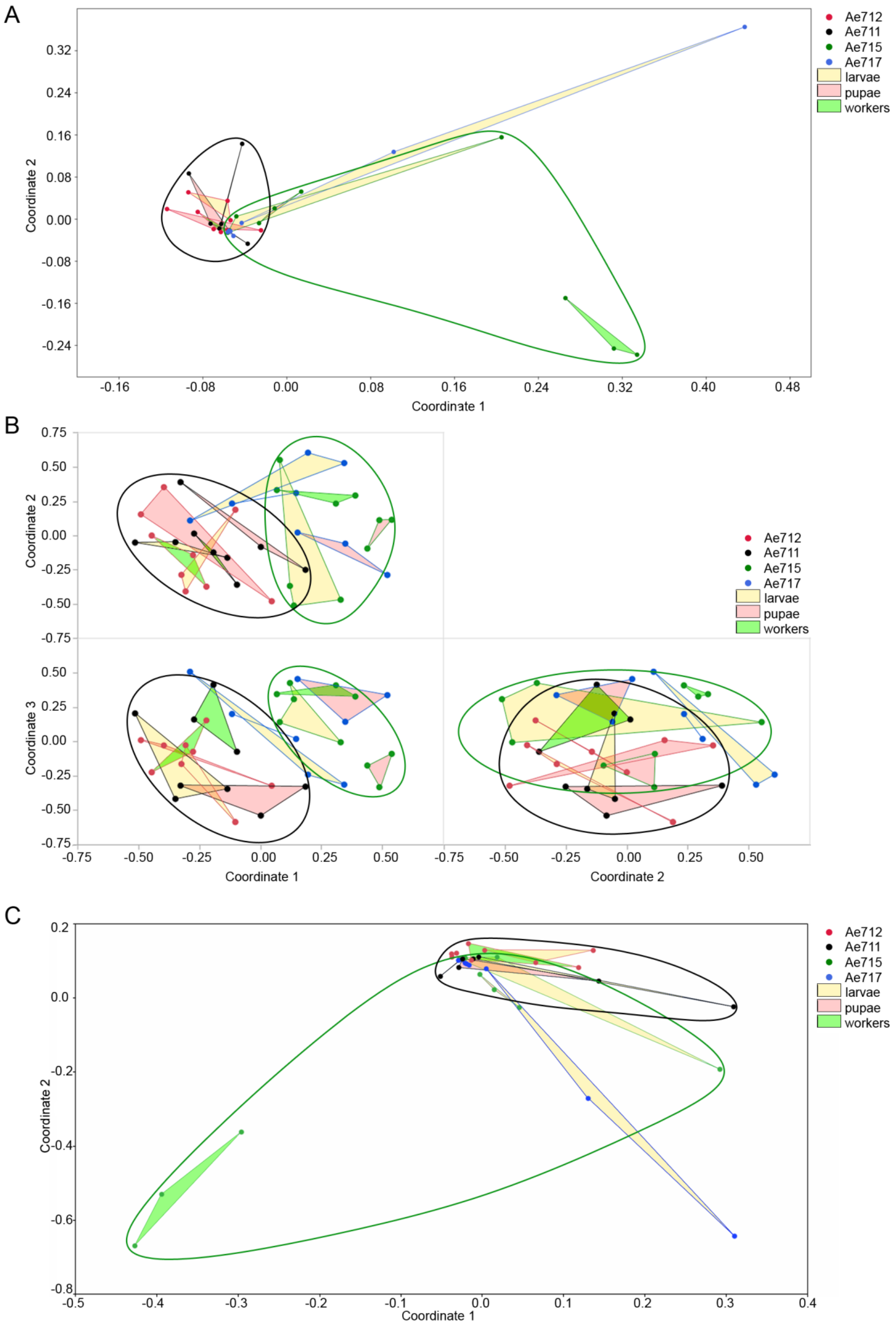
A
B
C
Ae712
Ae711
Ae715
Ae717
larvae
pupae
workers
Coordinate 1
Coordinate 2
Coordinate 3
0.32
0.24
0.16
0.08
0.00
-0.08
-0.16
-0.24
0.40
0.48
0.75
0.50
0.25
-0.25
-0.50
-0.75
0.2
0.0
-0.2
-0.4
-0.6
-0.8
-0.5
-0.3
-0.1
0.1
0.3
0.4

Figure S4. NMDS diagram of gut bacterial communities at different developmental stages of *A. echinatior* based on Bray-Curtis dissimilarities (A), unweighted and weighted UniFrac distances (B and C, respectively). The black outlines surround individuals of two colonies with dominant *Wolbachia* infection (Ae711 – black dots, Ae712 – red dots). The green lines delineate individuals of the colony (Ae715 – green dots) that was strongly infected with Mollicutes and also harboured *Wolbachia*. Blue dots represent individuals of *Wolbachia*-infected colony Ae717, which had a higher diversity of bacteria in its larvae. Connected dots represent bacterial communities of individuals at specific developmental stages for each colony: larvae, mature pupae and adult workers.

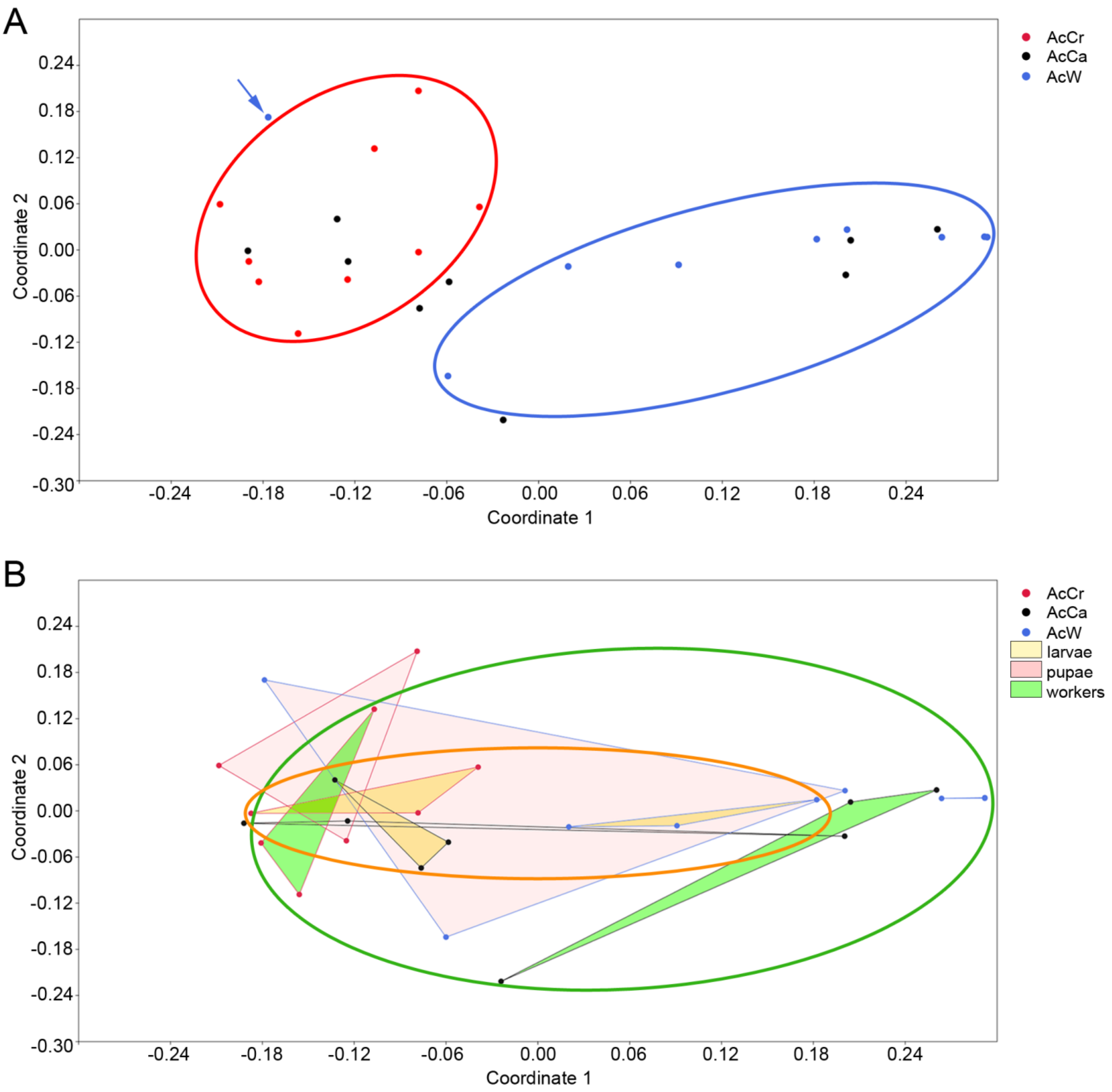

Figure S5. NMDS of Bray-Curtis dissimilarity distances using the rarefied OTU reads of gut bacteria at different developmental stages of *A. cephalotes*. (A) Ovals delineate the significantly different strongly Mollicutes-infected colony AcW (blue dots and oval) and the Mollicutes-uninfected colony AcCr (red dots and oval). A single Mollicutes-uninfected outlier from the AcW colony is highlighted with an arrow. The moderately Mollicutes-infected colony AcCa (black dots) had both Mollicutes-infected and Mollicutes-uninfected individuals. **(B)** Triangles represent bacterial communities of individuals at specific stages of development for each colony: larvae, mature pupae and adult workers. Significant differences between gut communities in larvae and workers are highlighted by ovals of corresponding colors.

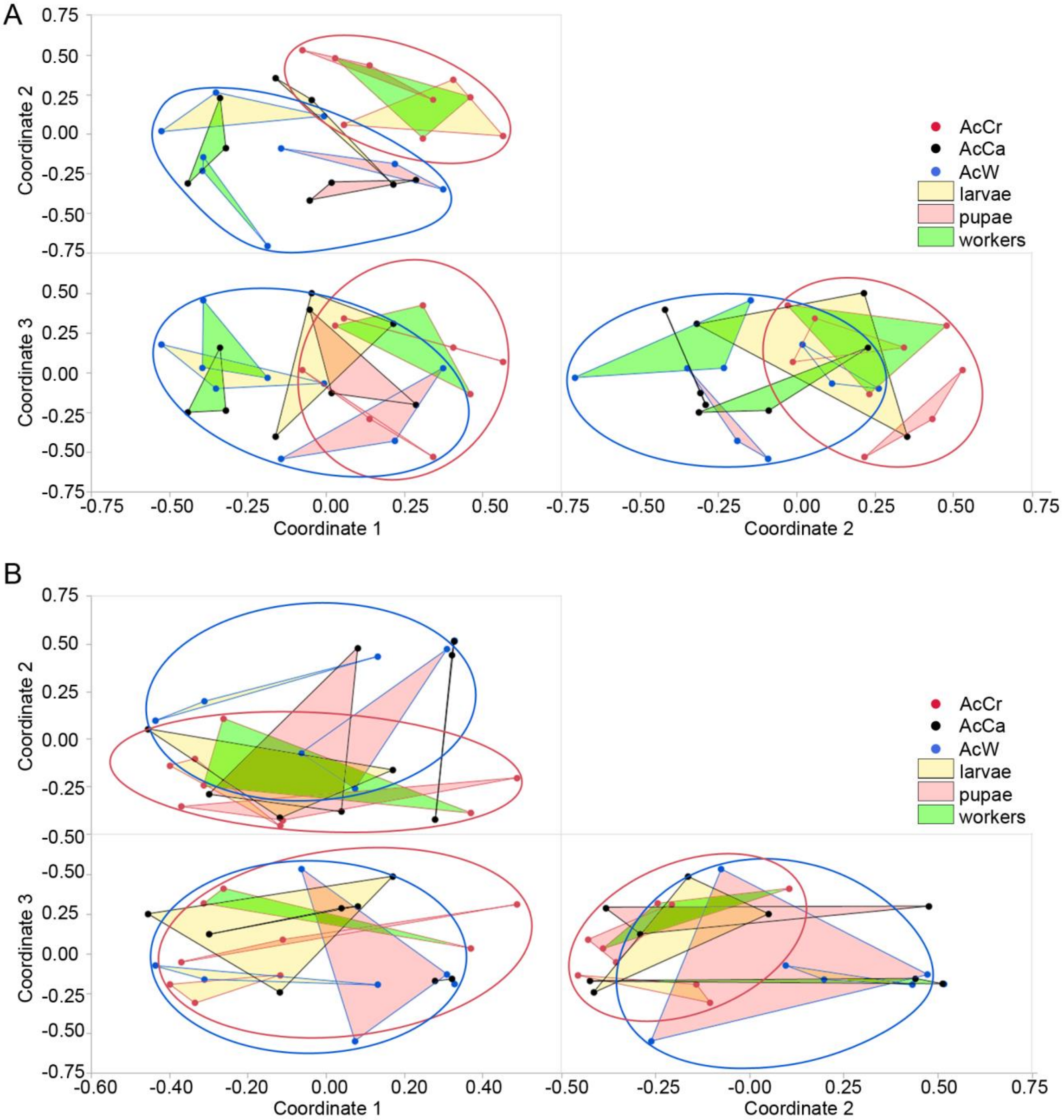

Figure S6. Plots showing the three-dimensional solution of NMDS of unweighted (A) and weighted UniFrac metrics (B) using the rarefied OTU reads of gut bacteria at different developmental stages of *A. cephalotes*. Ovals delineate the strongly Mollicutes-infected colony AcW (blue dots and oval) the Mollicutes-uninfected colony AcCr (red dots and oval), but the difference between them was not significant (see text). Triangles represent bacterial communities of individuals at specific stages of development for each colony: larvae, mature pupae and adult workers.

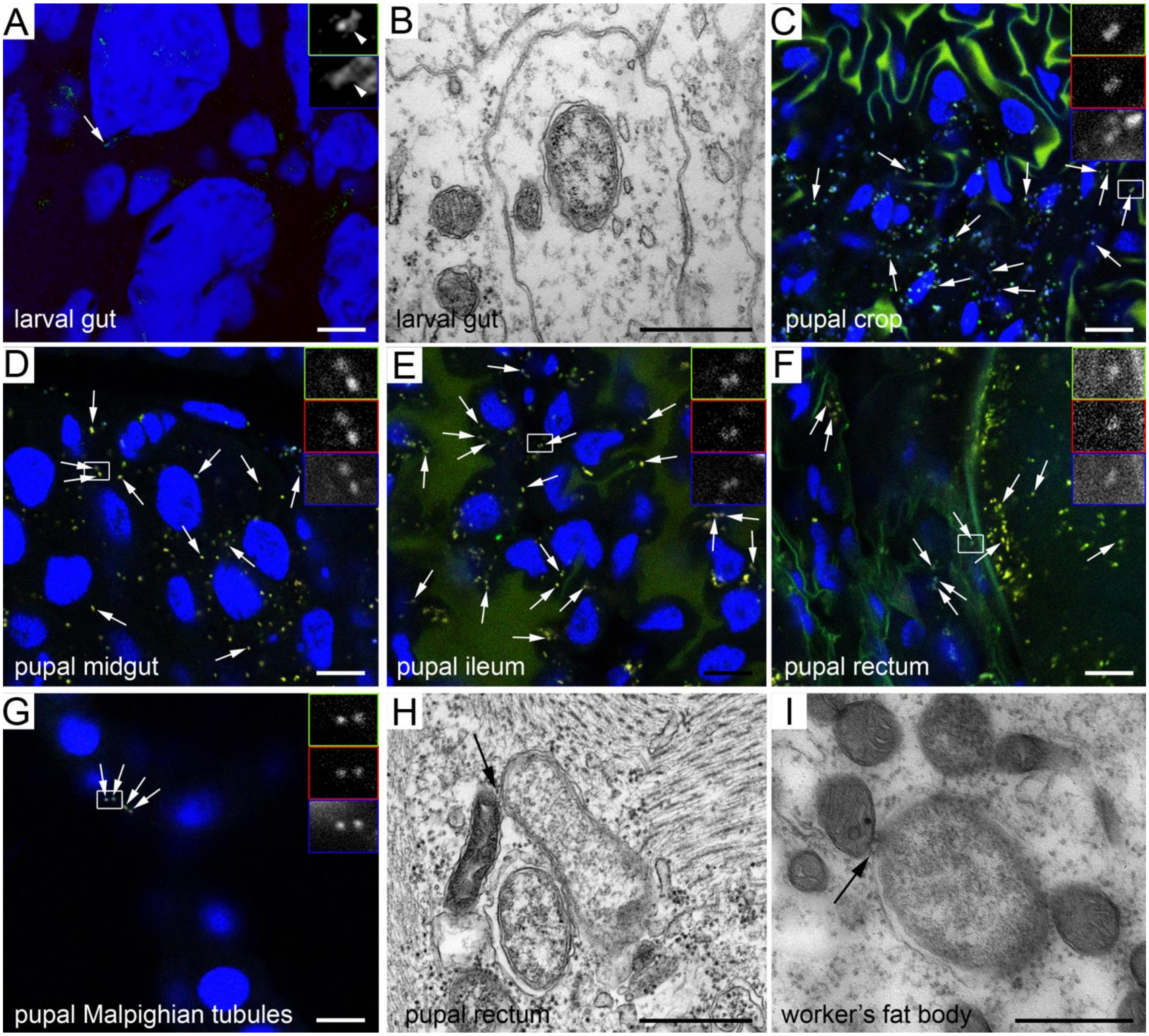

Figure S7. *Wolbachia* localization in *A. echinatior* for different developmental stages. (A, B) *Wolbachia* in epithelial cells of the larval gut in *A. echinatior*. **(C-G)** *Wolbachia* in different compartments of the pupal gut: the crop **(C),** the midgut **(D),** the ileum **(E)**, the rectum (**F**) and Malpighian tubules **(G). (H, I)** *Wolbachia* bacteria tightly interacting with mitochondria (black arrows) in a muscle cell of a pupa **(H)** and adult worker **(I)**. White arrows point at bacteria. The inserts show an overlapping staining with a *Wolbachia*-specific probes (framed green), an eubacterial probe (framed red, for pupae only) and DNA staining with DAPI (framed blue) at higher magnification. Scale bars are 10 µm **(A, C-G)** and 0.5 µm **(B, H, I)**.

Table S1. Primers and FISH probes used in the present study.

Target	PCR primers	Sequence	Annealing temperature, PCR/ddPCR	Fluorescence threshold
Mollicutes (*EntAcro1*, *EntAcro2*)	Entom_F	CTCTGTTGTAAGGGAAGAA	52-55°C	
EntAcro1/Mollicutes	Entom_A_R	CTGTCACATTCTAGTCGTAT	53°C/52°C	12000
EntAcro2/Mollicutes	Entom_B_R	TCTCTTGTATTCTAGTGATGT	55°C/53°C	10000
Eubacterial 16S rRNA	63F	GCAGGCCTAACACATGCAAGTC	-/60°C	12500
	355R	CTGCTGCCTCCCGTAGGAGT	-/60°C	
tbp (*A. echinatior*, *A. cephalotes*)	AeTBP-F1	AGGTTTGCGGCTGTTATCAT	-/60°C	9000
	AeTBP-R1	TTCTTGCGTACTTTCTGGCA	-/60°C	

Target	FISH probes	Sequence	Fluorochrome
Mollicutes (*EntAcro1*, *EntAcro2*)	Entom_A488	TGTTGTAAGGGAAGAA	Alexa488
Wolbachia	Wolbachia16SR	CAGATTTGAACCAGATAGA	Cy3
	Wolbachia W2	CTTCTGTGAGTACCGTCATTATC	Cy3
EUB388	all bacteria	GCTGCCTCCCGTAGGAGT	Cy5
non-EntomA	-	TATGCTGATCTTACACTGTC	Cy3

Table S3. Statistical comparisons of gut bacterial community structure at different developmental stages of *A. echinatior* and *A. cephalotes* (two-way PERMANOVA). Values in bold are statistically significant.

	Bray-Curtis dissimilarity			unweighted UniFrac distances			weighted UniFrac distances		
	**df**	**F**	**p**	**df**	**F**	**p**	**df**	**F**	**p**
Species comparison:									
Acromyrmex-Atta		45.67	**0.0001**		3.573	**0.0001**		32.19	**0.0001**
A. echinatior									
stage	2	5.24	**0.0007**	2	2.03	**0.0001**	2	6.03	**0.0006**
colony	3	4.83	**0.0002**	3	2.74	**0.0001**	3	5.79	**0.0005**
stage x colony	6	4.91	**0.0003**	6	1.4	**0.0007**	6	5.86	**0.0004**
A. cephalotes									
stage	2	2.63	**0.0028**	2	1.42	**0.0097**	2	2.84	**0.0111**
colony	2	2.48	**0.0037**	2	1.69	**0.0006**	2	3.19	**0.006**
stage x colony	4	1.19	0.2066	4	1.29	**0.0119**	4	1.18	0.266

Table S4. Statistical comparisons of gut bacterial communities between developmental stages and colonies of *A. cephalotes* (one-way PERMANOVA). Values in bold are statistically significant.

**Bray-Curtis dissimilarity**					
	**F**	**p**		**F**	**p**
stages	2.121	**0.0125**	colonies	2.922	**0.009**
	**p, Bonferroni-corrected values**			**p, Bonferroni-corrected values**	
larvae-pupae	0.1146		AcCa-AcCr	0.167	
larvae-workers	**0.0291**		AcCa-AcW	0.4833	
pupae-workers	0.2358		AcCr-AcW	**0.0021**	
**weighted UniFrac distances**					
	**F**	**p**		**F**	**p**
stages	2.34	**0.025**	colonies	2.69	**0.0125**
	**p, Bonferroni-corrected values**			**p, Bonferroni-corrected values**	
larvae-pupae	0.904		AcCa-AcCr	0.14	
larvae-workers	0.94		AcCa-AcW	0.99	
pupae-workers	0.98		AcCr-AcW	1	

Table S5. Presence of *Wolbachia* bacteria in lab colonies of *A. cephalotes* and *A. echinatior* based on PCR analysis. Numbers (1-3) represent individual ant samples. + – bacteria were present, **-** – bacteria were absent, n/a – not analysed.

**Ant colony**	**eggs**	**larval guts**			**pupal guts**			**pupal robs**			**worker's guts**		
		**1**	**2**	**3**	**1**	**2**	**3**	**1**	**2**	**3**	**1**	**2**	**3**
Ae322	+	+	+	+	+	+	+	+	+	+	+	+	+
Ae360	+	+	+	+	+	+	-	+	+	+	+	+	+
Ae507	+	+	+	+	+	+	+	+	+	+	+	+	+
Ac2012-2	-	n/a	n/a	n/a	n/a	n/a	n/a	n/a	n/a	n/a	-	-	-
Ac2012-3	-	n/a	n/a	n/a	n/a	n/a	n/a	n/a	n/a	n/a	-	-	-
Ac2012-4	-	n/a	n/a	n/a	n/a	n/a	n/a	n/a	n/a	n/a	-	-	-
Ac19BB	-	n/a	n/a	n/a	n/a	n/a	n/a	n/a	n/a	n/a	-	-	-

Table S6. Mollicutes infection status in the guts of larvae, pupae and workers sampled from lab and field colonies of *A. cephalotes* and *A. echinatior*. Data for lab colonies are based on PCR analysis and for field colonies on results of 16S rRNA gene sequencing. Numbers (1-5) represent individual ant samples, with + indicating bacteria were present, **-** indicating bacteria were absent, and n/a indicating not analysed. For the 16S rRNA gene sequencing data, only individuals with relative abundances of Mollicutes > 0.05% were considered as to be +.

Lab colonies

**Ant colony**	**larval guts**					**pupal guts**					**worker's guts**					**Bacterial OTU**
	**1**	**2**	**3**	**4**	**5**	**1**	**2**	**3**	**4**	**5**	**1**	**2**	**3**	**4**	**5**	
Ac 2012-2	-	-	-	+	-	-	-	-	-	-	+	+	+	+	+	*EntAcro1*
	-	-	-	-	-	-	-	-	-	-	-	-	-	-	+	*EntAcro2*
Ac 2012-3	n/a	n/a	n/a	n/a	n/a	n/a	n/a	n/a	n/a	n/a	-	-	-	-	-	*EntAcro1*
	-	-	-	-	-	-	-	-	-	-	-	+	+	+	+	*EntAcro2*
Ac 2012-4	-	-	-	-	-	-	-	-	-	-	-	+	+	+	+	*EntAcro1*
	-	-	-	-	-	-	-	-	-	-	+	+	-	+	+	*EntAcro2*
Ac19BB	n/a	n/a	n/a	n/a	n/a	n/a	n/a	n/a	n/a	n/a	-	-	-	-	-	*EntAcro1*
	-	-	-	-	-	-	-	-	-	-	+	+	+	+	+	*EntAcro2*
Ae322	+	+	+	-	-	-	-	-	-	+	-	+	+	-	+	*EntAcro1*
	-	-	-	-	-	-	-	-	-	-	+	+	+	+	+	*EntAcro2*
Ae360	+	-	+	-	-	-	-	-	-	-	+	+	+	+	+	*EntAcro1*
	-	-	-	-	-	-	-	-	-	-	-	-	-	+	-	*EntAcro2*
Ae507	-	+	+	-	+	-	-	-	-	-	+	+	+	+	+	*EntAcro1*
	n/a	n/a	n/a	n/a	n/a	n/a	n/a	n/a	n/a	n/a	-	-	-	-	-	*EntAcro2*

Field colonies

**Ant colony**	**larval guts**				**pupal guts**			**worker's guts**			**Bacterial OTU**
	**1**	**2**	**3**	**4**	**1**	**2**	**3**	**1**	**2**	**3**	
AcCa	-	+	-		+	-	-	+	+	+	*EntAcro1*
AcCr	-	-	-		-	-	-	-	-	-	*EntAcro1*
AcW	+	+	+		-	+	+	+	+	+	*EntAcro1*
Ae711	-	-	-		-	-	-	+	-	-	*EntAcro1*
Ae712	-	-	-		+	-	-	-	-	-	*EntAcro1*
Ae715	-	-	+	-	-	-	-	+	+	+	*EntAcro1*
Ae717	-	-	-		-	+	-	-	-		*EntAcro1*

Table S7. Presence of two Mollicutes OTUs (*EntAcro1* and *EntAcro2*) in pupal guts and the rest of the bodies of the same individuals of *A. echinatior*. Each cell represents one individual ant sample.

Pupae	Field colonies												Bacterial OTU
	Ae711			Ae712			Ae715			Ae717			
Gut	-	-	-	-	-	-	-	-	-	-	-	-	*EntAcro1*
Rest of the body	-	-	-	-	-	-	-	-	-	-	-	-	
gut	-	-	-	-	-	-	-	-	-	-	-	-	*EntAcro2*
Rest of the body	-	-	-	-	-	-	-	-	-	-	-	-	

Pupae	Lab colonies															Bacterial OTU
	Ae322					Ae360					Ae507					
Gut	-	-	-	-	-	-	-	-	-	-	-	-	-	-	-	*EntAcro1*
Rest of the body	-	-	-	-	-	-	-	+	-	-	+	+	+	+	+	
Gut	-	-	-	-	-	-	-	-	-	-	-	-	-	-	-	*EntAcro2*
Rest of the body	+	+	+	+	-	+	+	+	+	+	-	-	-	-	-	

Table S8. Infection of workers with *EntAcro1* and *EntAcro2* OTUs before the transinfection experiment and 9 month after the first sampling (Table 1). + indicates that bacteria were present, (+) that bacteria were present only in a very low abundance, corresponding to extremely faint bands in agarose gel electrophoresis; **-** that bacteria were absent.

**Ant colony**	***A. echinatior* 360**					***A. echinatior* 322**					***A. echinatior* 507**					***A. cephalotes* 2012-2**					***A. cephalotes* 2012-3**				
**OTU**	**1**	**2**	**3**	**4**	**5**	**1**	**2**	**3**	**4**	**5**	**1**	**2**	**3**	**4**	**5**	**1**	**2**	**3**	**4**	**5**	**1**	**2**	**3**	**4**	**5**
EntAcro 1	+	+	+	+	+	(+)	(+)	(+)	+	+	+	+	+	+	+	+	+	+	+	+	+	+	+	+	+
EntAcro 2	-	-	-	-	-	+	+	+	+	+	+	-	-	+	-	-	-	+	-	-	-	+	+	-	-
